# Reduction of lithium induced interstitial fibrosis on co-administration with amiloride

**DOI:** 10.1038/s41598-022-18825-1

**Published:** 2022-08-26

**Authors:** Paulomi M. Mehta, Gregory Gimenez, Robert J. Walker, Tania L. Slatter

**Affiliations:** 1 Department of Pathology, Dunedin School of Medicine, University of Otago, Dunedin, New Zealand; 2 Department of Medicine, Dunedin School of Medicine, University of Otago, Dunedin, New Zealand

**Keywords:** Chronic kidney disease, Interstitial disease, Renal fibrosis, Diseases, Nephrology, Pathogenesis

## Abstract

Long-term administration of lithium is associated with chronic interstitial fibrosis that is partially reduced with exposure to amiloride. We examined potential pathways of how amiloride may reduce interstitial fibrosis. Amiloride was administered to a rat model of lithium induced interstitial fibrosis over a long term (6 months), as well as for short terms of 14 and 28 days. Kidney cortical tissue was subjected to RNA sequencing and microRNA expression analysis. Gene expression changes of interest were confirmed using immunohistochemistry on kidney tissue. Pathways identified by RNA sequencing of kidney tissue were related to ‘promoting inflammation’ for lithium and ‘reducing inflammation’ for amiloride. Validation of candidate genes found amiloride reduced inflammatory components induced by lithium including NF-κB/p65^Ser536^ and activated pAKT^Ser473^, and increased p53 mediated regulatory function through increased p21 in damaged tubular epithelial cells. Amiloride also reduced the amount of *Notch1* positive PDGFrβ pericytes and infiltrating CD3 cells in the interstitium. Thus, amiloride attenuates a multitude of pro-inflammatory components induced by lithium. This suggests amiloride could be repurposed as a possible anti-inflammatory, anti-fibrotic agent to prevent or reduce the development of chronic interstitial fibrosis.

## Introduction

Long-term administration of lithium to treat mood disorders can cause nephrogenic diabetes insipidus (NDI) and chronic interstitial fibrosis with tubular atrophy^1^. Many aspects of chronic kidney fibrosis are mimicked in a rat model of lithium induced interstitial fibrosis where six months of lithium treatment results in progressive interstitial fibrosis, increased myofibroblasts, enhanced TGFβ1, along with cystic dilation of the cortical collecting ducts^2–4^.

Exposure to lithium has demonstrated increased phosphorylation (inhibition) of glycogen synthase kinase 3β (GSK3β), decreased phosphorylation of β-catenin with an increase in cyclin D1 and activation of the Akt pathway^5–8^. Using single tubule RNA Seq of microdissected rat cortical collecting ducts following 72 h of lithium exposure demonstrated overexpression of a number of intracellular pathways including ‘cell cycle signaling’, ‘NF-κB signaling’, ‘p53 signaling’, ‘Wnt signalling’ and ‘aldosterone up-regulated genes’^9^. However, the focus of these investigations was on the mechanisms of lithium induced NDI and not on pathways leading to chronic interstitial fibrosis.

Short-term animal models of lithium exposure have demonstrated the development of dilated distal tubules with principal cell proliferation, with subsequent reversal of the ratio of principal to intercalated cells^6,10,11^. The proliferating cells are characterized by cell cycle arrest in G2M phase^6–8^. We have previously shown that amiloride can reverse lithium induced NDI and reduce the degree of fibrosis as demonstrated by reduced collagen deposition and myofibroblasts and significantly reduced expression of TGFβ1 and CTGF (connective tissue growth factor)^3^. Of note, amiloride did not modify the dilated distal tubules and altered tubular cell morphology induced by lithium^3^.

Against this background, we examined the interactions between lithium and amiloride following short-term exposure (14 and 28 days) and long-term exposure (amiloride commenced after 1 month of lithium exposure for total of 5 months) on intracellular signaling pathways using RNA Seq followed by in situ based analyses. We demonstrate that amiloride attenuates a multitude of pro-inflammatory pathways induced by lithium, suggesting that amiloride has additional anti-fibrotic and anti-inflammatory properties in addition to its action blocking ENaC activity.

## Results

### Long-term amiloride co-administration reduces fibrosis and inflammatory gene sets

**Figure 1:**
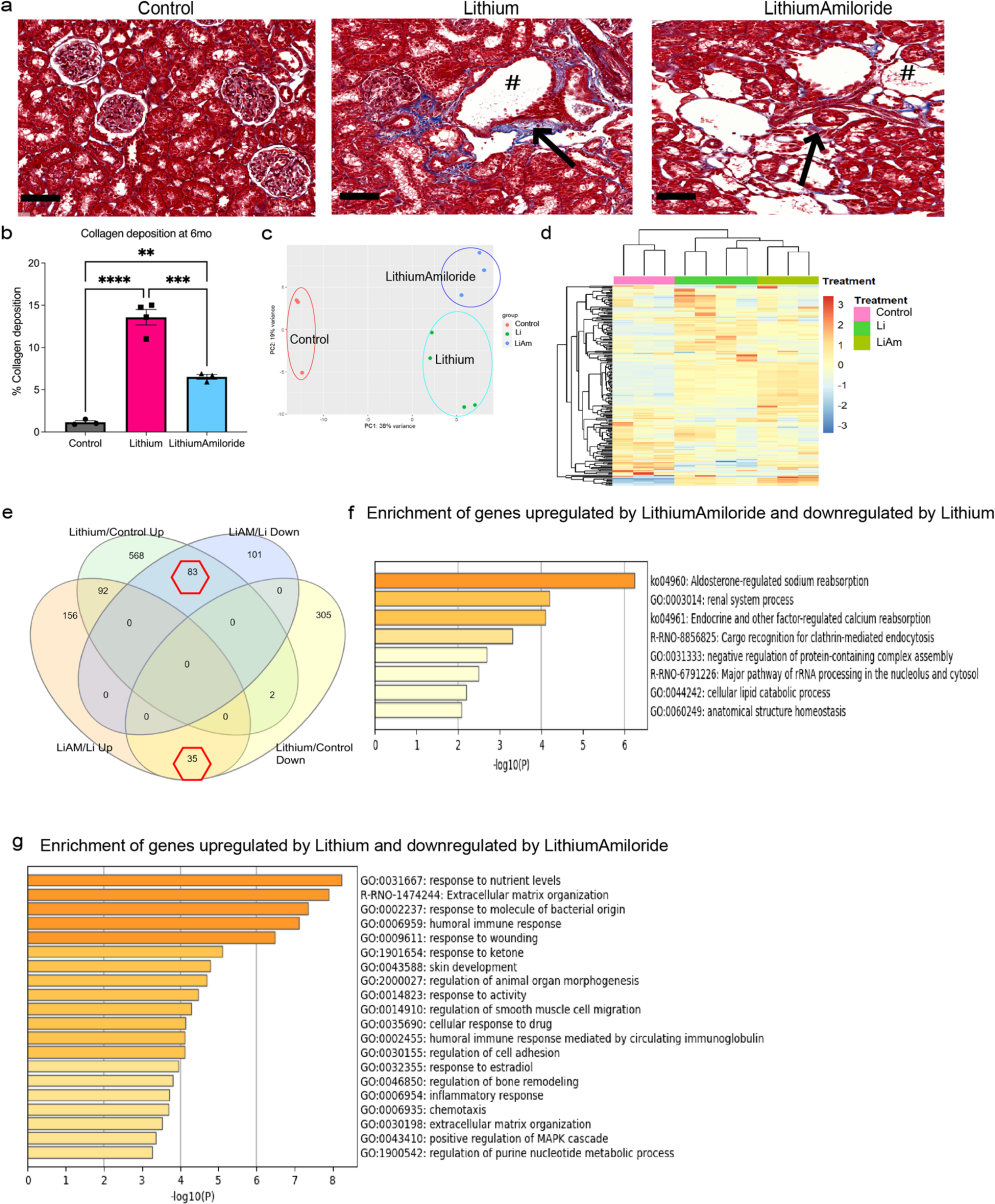
Amiloride exposure has distinct genetic changes with chronic lithium exposure. (**a**) Masson’s Trichrome staining of kidney tissue from control, lithium alone and Lithium and Amiloride treatments in the 6 month animal group. Arrows highlight regions with collagen (blue) deposition. # indicates dilated tubules. Scale bar 100 µm. (**b**) Quantification of collagen deposited. (**c**) Principal component analysis showing samples from the same group cluster together. Control (red), lithium alone (Li, green) and lithium and amiloride (LiAM, blue). (**d**) Top 200 most variable genes clustered samples into their distinct treatment. Shades of yellow–red indicate increased transcript abundances whereas shades of blue indicate decreases transcript abundances. (**e**) Venn diagram of transcripts increased or decreased with different comparison amongst treatment groups, also illustrating the number of common genes. (**f**) Enrichment of common genes increased by LithiumAmiloride and decreased by lithium. (**g**) Enrichment of common genes increased by Lithium and decreased by LithiumAmiloride. Significance **P < 0.01, ***P < 0.001, ****P < 0.0001. *ns* not significant. Data represented as mean ± sem.

We previously showed that five months of co-administered amiloride therapy in an animal model of lithium induced CKD significantly reduced interstitial fibrosis^3^ (Fig. 1a,b). Principal component analysis (PCA) on transformed RNA Seq data showed most samples clustered within their respective treatment groups, suggesting gene expression changes were treatment related (Fig. 1c,d). Samples that did not cluster with the respective group were eliminated (Supplementary Fig. S1a).

Analysis of differentially expressed genes is given in Supplementary Dataset 1.

Overrepresentation analysis of common genes that were increased with Li/control but decreased in the LiAM/Li comparison (n = 83) were enriched in ‘response to nutrient levels’, ‘extracellular matrix organization’ and other ‘immune related pathways’ (Fig. 1e,f). Not unexpectedly, pathways enriched in the ‘Aldosterone regulated sodium reabsorption’, ‘renal system processes’, and ‘endocrine and other factor-regulated calcium reabsorption’ were increased with LiAM/Li but decreased in the Li/Control comparison (n = 35) (Fig. 1e,g). In the LiAM/control comparison, ontologies overrepresented amongst decreased genes (n = 386) were enriched in ‘extracellular matrix organization’ and ‘response to nutrient levels’ amongst others and ontologies with increased genes in the same group (n = 838) were enriched in ‘epithelial cell differentiation’, ‘metal ion transport’, ‘aldosterone-regulated sodium reabsorption (Supplementary Dataset 2a).

Given that amiloride co-administration had reduced interstitial fibrosis, we focused on genes related to inflammation and fibrosis pathways, that were increased by lithium and decreased by amiloride. Network plot of the differentially expressed genes from the Li/control demonstrate increased pro-inflammatory and pro-fibrotic genes (Supplementary Fig. S2a). In contrast the network plot of LiAM/Li comparison showed decreased expression of pro-fibrotic and inflammatory genes (Supplementary Fig. S2b).

### Reduced fibrosis and inflammatory signatures with amiloride exposure are evident in the short-term

The results of our long-term amiloride exposure suggested amiloride had anti-inflammatory and anti-fibrotic properties. We next determined if these changes occurred with early exposure to amiloride administered concurrently with lithium from day zero, using shorter treatment periods of 14 and 28 days. As expected, the lithium animals all demonstrated NDI which was reduced in the amiloride group (Supplementary Table 1).

**Figure 2:**
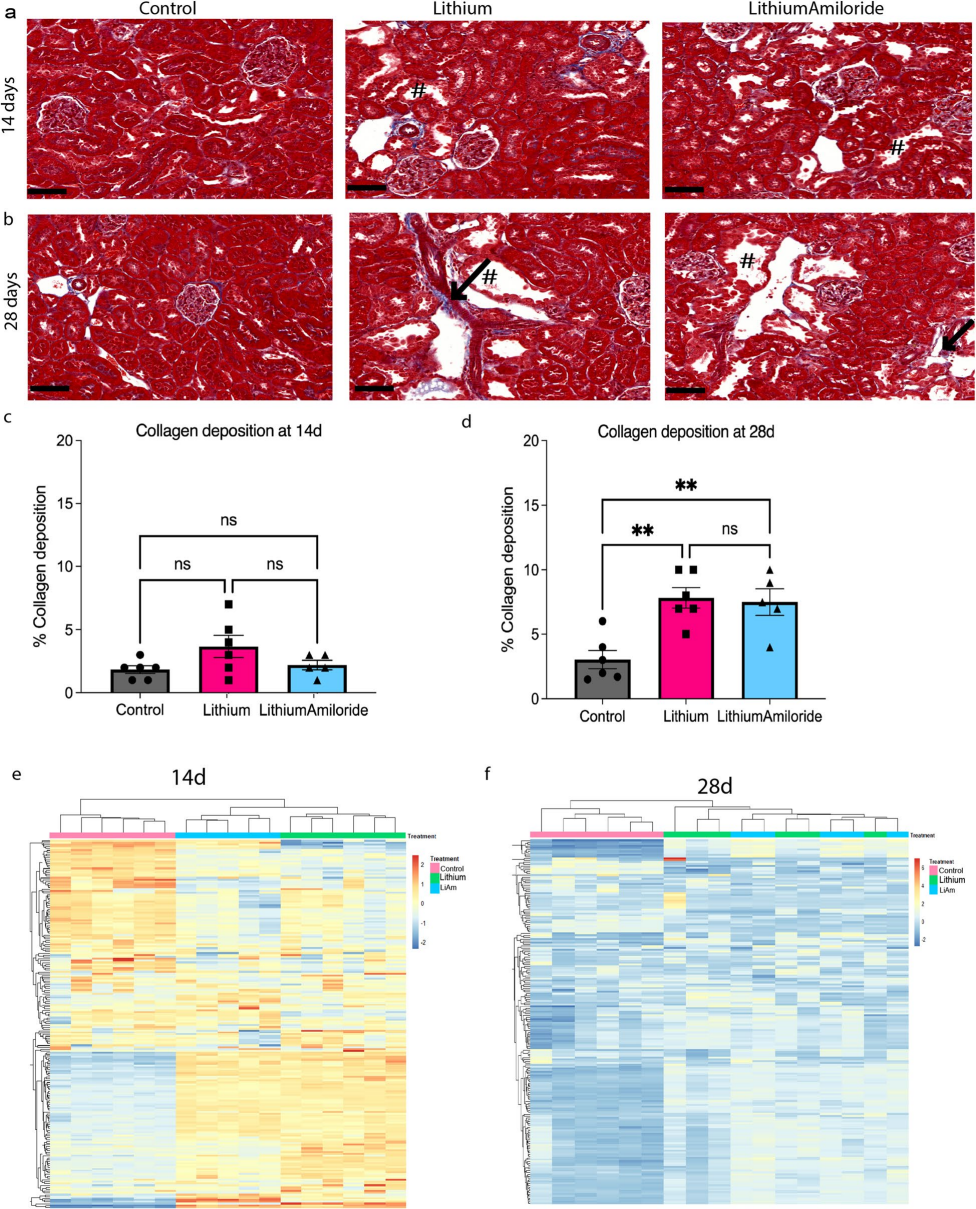
Amiloride exposure has distinct genetic changes with 14, but not 28 days of exposure. (**a**, **b**) Masson’s Trichrome staining of kidney tissue from control, lithium alone and Lithium and Amiloride treatments for 14 or 28 days. Arrows highlight regions with collagen (blue) deposition. ^#^Indicates dilated tubules. Scale bar 100 µm. (**c**, **d**) Quantification of collagen deposited, (**e**, **f**) Top 200 most variable genes clustered samples into their distinct treatment groups at 14, but not at 28 days. Shades of yellow–red indicate increased transcript abundances whereas shades of blue indicate decreases transcript abundances. Significance **P < 0.01, *ns* not significant. Data represented as mean ± sem.

Following 14 days of lithium treatment alone or combined with amiloride, the characteristic morphological changes of distal tubular cystic dilations (Fig. 2a) were evident. There was some interstitial fibrosis evident, but this did not reach significance compared to controls (Fig. 2c). After 28 days of treatment, the same morphological changes were visible to a greater extent in both the lithium and lithium-amiloride groups (Fig. 2b). Interstitial collagen deposition was increased in the lithium alone group to compared to control and lithium-amiloride groups at 28 days (Fig. 2d).

RNA Seq performed on kidney cortical tissue from all 14- and 28-day treatment groups showed most of the 14-day samples (except one) clustered within their respective treatment in the Principal Component Analysis (Supplementary Fig. S1b). The heatmap of the most variable top 200 gene counts suggested the gene expression changes were treatment related (Fig. 2e). In the 28-day data only the control group clustered as a separate group, with the lithium and lithium-amiloride groups clustering together (Supplementary Fig. S1c). This was suggestive that actions of amiloride on modulating lithium induced gene expression may vary over this time course. (Fig. 2f).

Analysis of significantly differential gene expression is given in Supplementary Dataset 1.

In line with studies over a shorter time frame^9^, ontologies of differentially expressed genes increased with lithium administration at 14 days (n = 1264) were enriched for ‘inflammatory response’, ‘lymphocyte activation’, ‘innate immune response’ and ‘cytokine-mediated signalling pathway’ (Supplementary Dataset 2b). Specific genes differentially expressed in the lithium group correlating with the inflammatory response included *Nfkb2* (0.76log2FC), subunit of NF-κB (Supplementary Dataset 1 and Supplementary Fig. S3a). These also included genes that encode T cell receptor *CD3* (*cd3d*, 1.92, *cd3e*, 1.64 and *cd3g*, 1.54log2FC), *CD8* cytotoxic T cell (*cd8b*, 1.13 and *cd8a*, 0.69log2FC), along with activator Z*ap70* (1.40log2FC) and *fos* (3.48log2FC) important for activation and differentiation of leukocytes^12^. Other significantly expressed genes were targets of tumor suppressor protein 53 (p53) including *cdkn1a*/p21 (4.14log2FC), and *cyclin D1* (0.91log2FC) and *tert* (0.71log2FC), which are associated with epithelial cell proliferation^13–15^. Of interest, GSK3β gene expression was not differentially altered, despite the key role this kinase plays in mediating the observed changes induced by lithium.

We focused on the genes modulating inflammation, cellular proliferation and fibrosis. Overrepresentation analysis of common genes increased with lithium treatment and decreased in response to lithium-amiloride at 14 days (n = 218), showed lithium induced positive enrichment of ‘inflammatory response’, ‘cytokine production’, ‘cell adhesion’, ‘innate immune response’ and ‘wound healing’ gene sets as previously reported^9^ (Supplementary Fig. S4b). Consistent with data at six-months, ‘Aldosterone-regulated sodium reabsorption’ was overrepresented amongst common genes decreased by lithium and increased with lithium-amiloride (n = 43) (Supplementary Fig. S4C).

At 28 days, lithium induced increased genes were overrepresented in ‘Cell adhesion molecules (CAMs)’ and ‘inflammatory response’ signalling pathway. Lithium-amiloride had ‘Aldosterone-regulated sodium reabsorption’ pathway overrepresented amongst increased genes (Supplementary Dataset 2b). Here there were fewer differential and common genes (n = 19) consistent with the groups clustering together (Fig. 2f). These common genes were enriched in response to nutrient levels and wound healing amongst others (Supplementary Fig. S4e). Many of the significantly differentially expressed genes from the Li/Control and LiAM/Li comparisons at six months were altered in the 14-day data. The pro-fibrotic and inflammatory genes decreased in the LiAM/Li comparison at both 6 months and 14 days included *serpine1*, *osmr*, *dll1*, *socs3*, *myc*, *angptl4*, *lamb3*, *havcr1* and metalloproteinase genes. Short-term treatment demonstrated that amiloride produced a reduction in the anti-inflammatory gene signatures before a significant difference in interstitial fibrosis was evident.

### Reduced inflammation and T-cell infiltration with amiloride exposure

**Figure 3:**
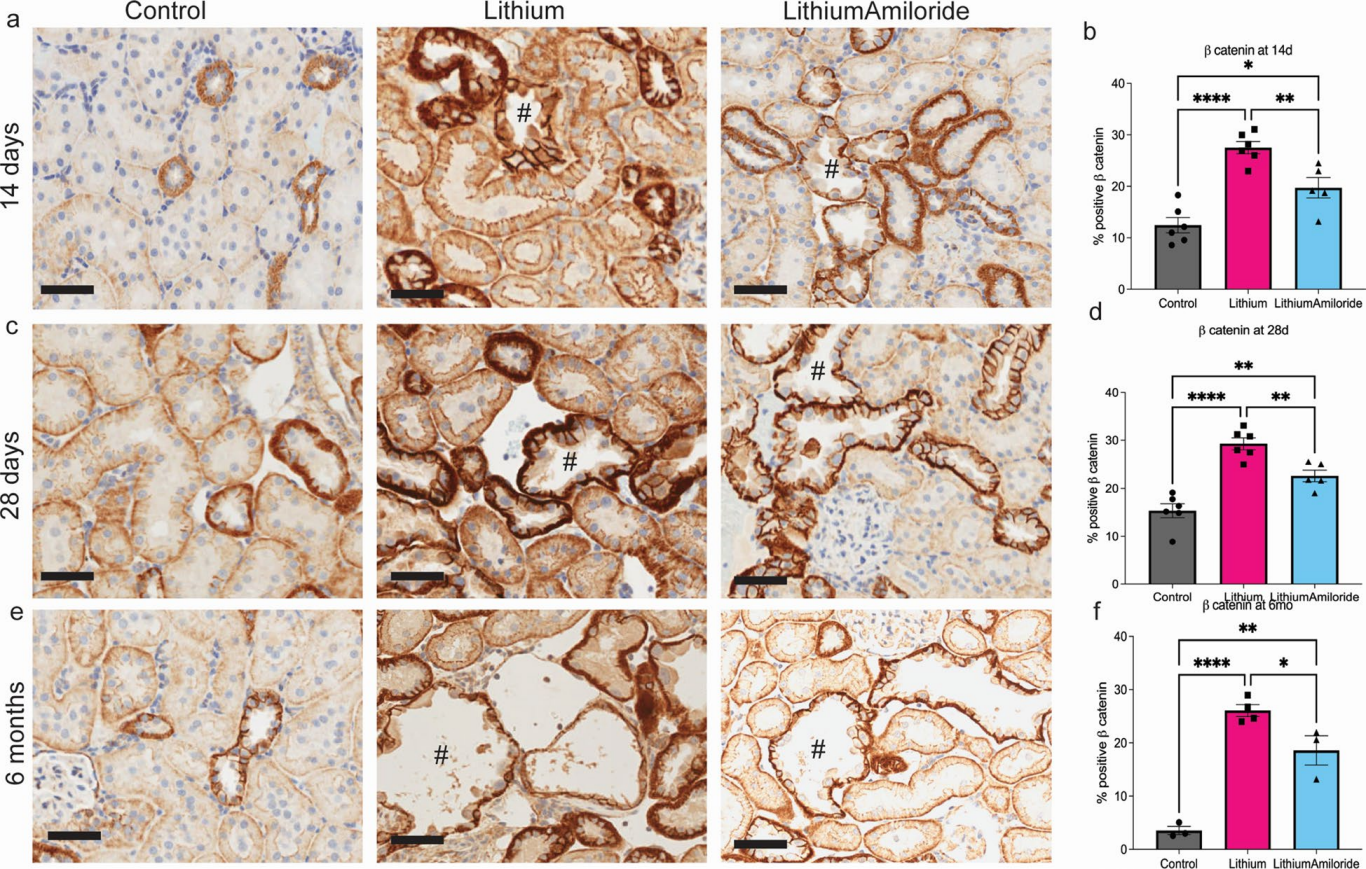
Lithium treatment increases β-catenin at all time points. Representative images of β-catenin immunohistochemistry staining and quantification for. (**a**, **b**) 14 days; (**c**, **d**) 28 days and (**e**, **f**) 6 months. Intense brown staining show β-catenin positive cells. ^#^Indicates dilated tubules. Scale bar 50 µm Significance * *P* < 0.05, ** *P* < 0.01, ****P < 0.0001. Data represented as mean ± sem.

The RNA Seq analyses suggested amiloride reduces multiple facets of lithium induced inflammation and T-cell mediated immune response. NF-κB a key inflammatory pathway can be positively and negatively modulated by Wnt/βcatenin signalling^16^ and negatively regulated by p53 signalling^17^. The Wnt/βcatenin pathway is regulated by GSK3β, a known target for lithium^18,19^. Therefore, we first investigated β-catenin expression, which had increased nuclear translocation in the tubular epithelial cells in the lithium samples, but was significantly reduced in the lithium amiloride group (Fig. 3).

**Figure 4:**
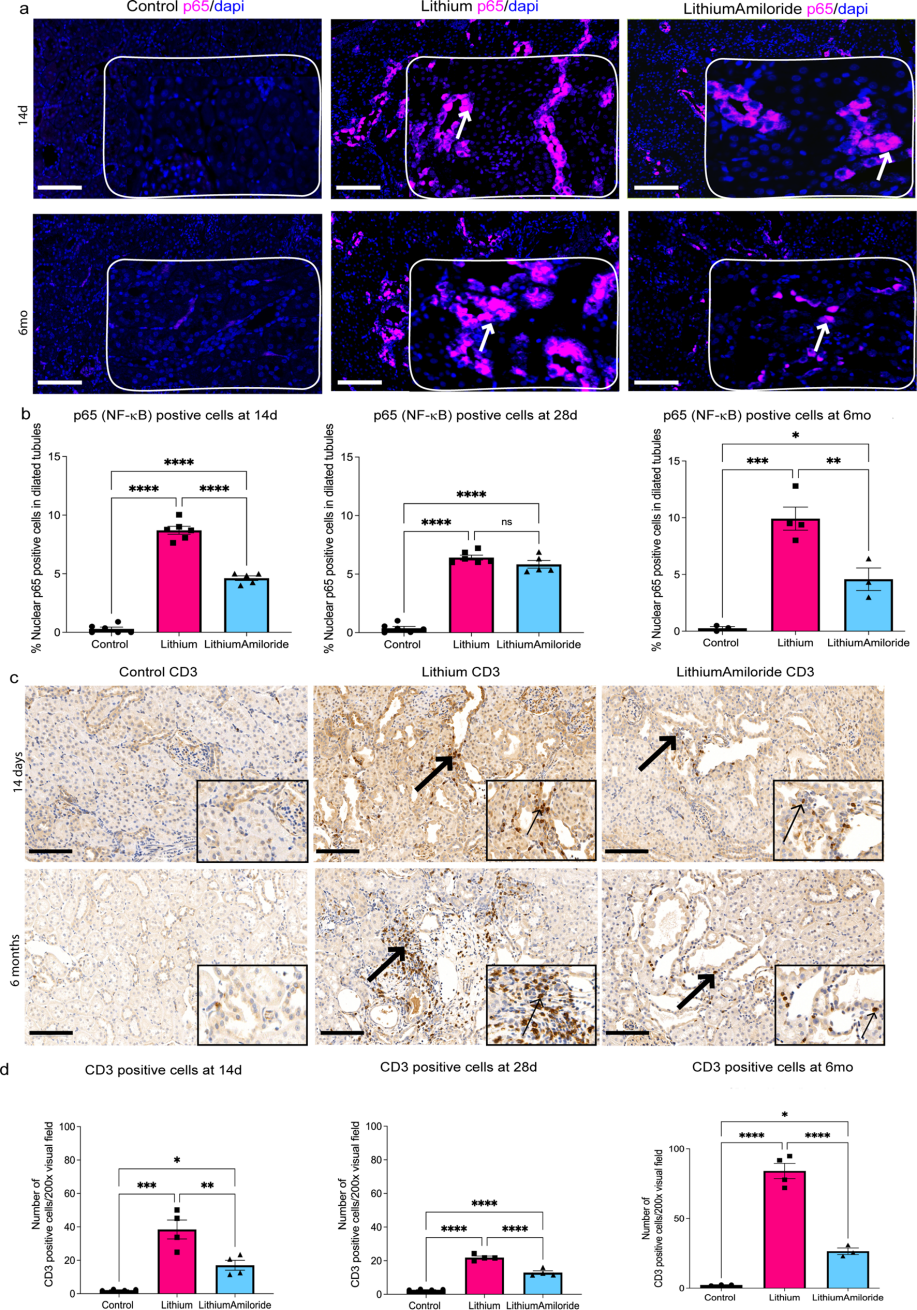
Amiloride exposure reduces inflammation early and late in the course of lithium treatment. (**a**) Nuclear factor kappa B cell (NF-κB)/ p65 (pink) staining in kidney tissue from control (top), lithium (middle) and animals treated with both lithium and amiloride (LithiumAmiloride) (bottom). p65/NF-κB positive cells highlighted with arrows. (**b**) Quantification of nuclear p65/NF-κB in tubular cells at 14 days, 28 days and 6 months. (**c**) Representative images of CD3 positive cells (black arrow) across all treatment groups at 14 days and 6 months. (**d**) Quantification of the average number of CD3 positive cells from 10 visual fields at magnification of 200 ×. Scale bar 80 µm, inserts at 25 µm Significance *P < 0.05, **P < 0.01, ***P < 0.001, ****P < 0.0001. ns- not significant. Data represented as mean ± sem. Representative images for p65 at 28 days are given in Supplementary Fig. S6b.

We next investigated if the inflammatory marker nuclear p65^ser536^/RelA, a subunit of the NF-κB complex was differentially expressed between treatment groups. Increased nuclear p65^ser536^ staining was found in the dilated tubules, and a higher percentage of p65^ser536^ positive cells was found at 14 and 28 days, and six months in the lithium and lithium-amiloride groups compared to controls (Fig. 4a,b). The lithium-amiloride group at 14 days and six months had reduced p65^ser536^ staining compared to the lithium group but this was still increased compared to controls. CD3 staining for T cells showed these cells were largely present in the interstitium (Fig. 4c). The lithium groups had the highest number of T cells with the largest amount evident in the six-month cohort (Fig. 4d). Lithium-amiloride significantly reduced the number of T cells at all time points, most evident at six months compared to lithium alone. The findings of reduced NF-κB signalling and T-cell infiltration is consistent with amiloride having profound anti-inflammatory effects in this model.

### Amiloride affects inflammatory regulators upstream of NF-κB signalling

**Figure 5:**
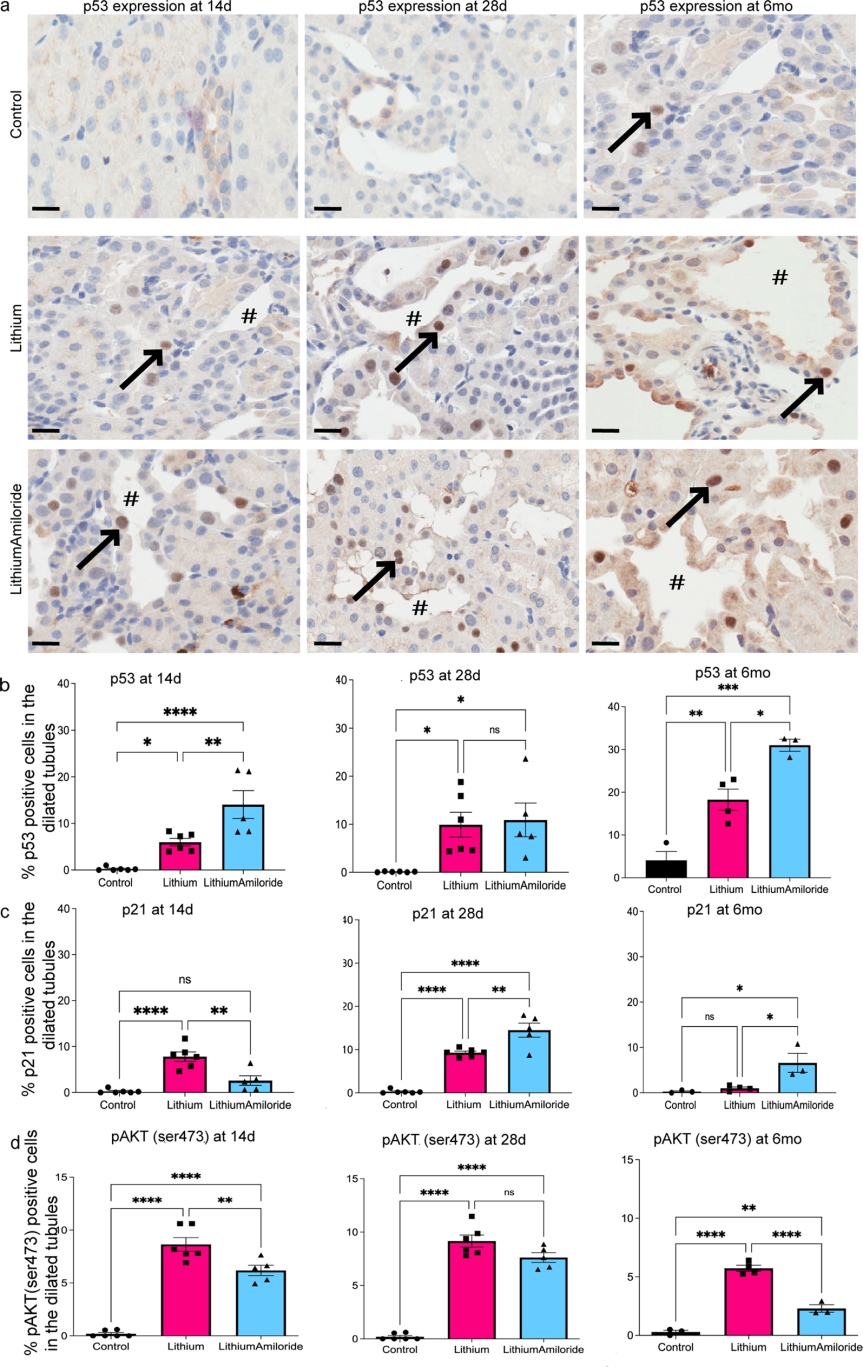
Amiloride exposure alters the amount of p53, p21 and pAKT in the kidney. (**a**) p53 protein detected using immunohistochemistry at 14 days, 28 days and 6 months in response to control, lithium only and LithiumAmiloride treatment. Arrows highlight positive staining in dilated tubules. #Indicate dilated tubules. Magnification of 400 ×. Scale bar 50 µm. (**b–d**) Quantification of percent positive cells using immunohistochemistry to detect p53, p21 and pAKT. Significance *P < 0.05, **P < 0.01, ***P < 0.001, ****P < 0.0001. *ns* not significant. Data represented as mean ± sem.

Fibrosis is a consequence of persistent tubular epithelial injury leading to maladaptive repair and inflammation^20^. RNA seq analysis highlighted *cdkn1a/p21*, a key target of p53 at all time points (1.25log2FC at six months, 3.19 log2FC at 28 days and 4.14log2FC at 14 days). Tumor suppressor p53 has a well-established role in responding to stress and preventing inflammation which may be modified with lithium and amiloride treatment^21,22^. To test if p53 was altered with lithium and/or amiloride exposure, p53 and its downstream target (p21), a measure of activated p53, were investigated using immunohistochemistry. p53 and p21 were almost exclusively found in the nuclei of cells in dilated distal tubules (Fig. 5a). In the lithium and lithium-amiloride treatment groups, p53 staining was increased at all time points compared to controls (Fig. 5b). The percentage of p53 staining in lithium induced dilated tubular cells was highest at six months and of interest, this was evident most in the lithium-amiloride group. Associated with this was an increased expression of miRNA34a which is known to be transactivated by p53^23^ (Supplementary Fig. S5). At 14 days, p53 staining was again highest in the lithium-amiloride group; however, no difference between the lithium-amiloride and lithium groups was found at 28 days (Fig. 5b). The p21 results largely followed the p53 results at six months and 28 days; however, p21 staining was highest in lithium treated animals at 14 days (Fig. 5c). These results suggest p53 responds to lithium induced cellular damage. The enhanced p53 response with amiloride exposure is consistent with p53 contributing to amiloride’s anti-inflammatory properties with reduced p65/NF-κB expression.

We next explored if amiloride’s effect on p53 would attenuate pro-inflammatory regulators that can function in the absence of p53, such as activated pAKT^ser473^ that can promote p53 degradation^24–27^. An analysis of pAKT^ser473^ expression in all treatment groups found pAKT^ser473^ was localised in the dilated tubular epithelial cells and was increased in lithium and lithium-amiloride groups compared to controls. At 14 days and 6 months pAKT^ser473^ expression was highest in lithium compared to the corresponding lithium-amiloride group (Fig. 5d). Again, at 28 days pAKT^ser473^ showed no difference between the lithium and lithium-amiloride groups (Fig. 5d). These results suggest amiloride reduces pAKT^ser473^ function supporting an anti-inflammatory role for amiloride. The lack of difference observed in the number of p65 and pAKT positive cells between lithium and the lithium-amiloride groups at 28 days in particular was interesting. While we do not fully understand this phenomenon, we propose a transition from acute injury between 14 and 28 days to chronic injury seen at 6 months. Specifically, it could be an acute inflammatory response to lithium at 14 days reduced by amiloride and a potential wound healing stage at 28 days where the effect of lithium and lithium-amiloride on inflammation equilibrate. Lower doses of lithium have been shown promote tubular epithelial cell recovery by directly inhibiting GSK3β, or indirectly via phosphorylating Akt. The short term beneficial effect of lithium at 14 days could be due to processes that are intrinsically linked with cell proliferation by upregulation of cell cycle kinases thereby preventing apoptosis with reduced fibrosis^28^.

### Amiloride co-administration reduces pericytes and *Notch1* accumulation in the interstitium

**Figure 6:**
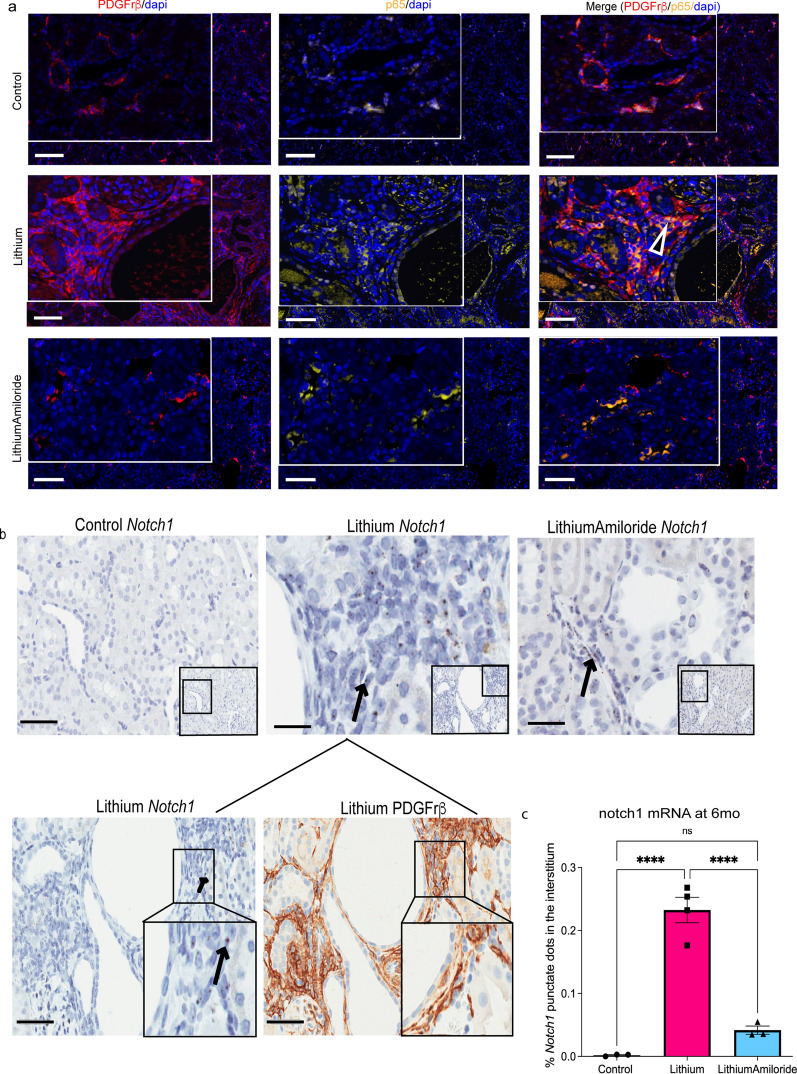
Reduced notch 1 positive pericytes in the kidney interstitium following amiloride exposure. (**a**) Nuclear factor kappa B cell (NF-κB)/p65 and PDGFrβ positive pericytes are decreased in the interstitium of the LithiumAmiloride compared to the lithium alone group in six-month animals. PDGFrβ positive pericytes (red), nucleus (blue), p65 (yellow), right, merge of PDGFrβ and p65. Arrowhead shows double positive cells. (**b**) *Notch 1* RNAscope in situ hybridization in the interstitium of animals treated with lithium alone and decreased in the LithiumAmiloride six-month animals. Brown punctate dot indicate positive signal of *Notch1* gene expression. Notch 1 positivity was found in regions with PDGFrβ positive cells. Images taken at 400 × magnification with a scale bar of 50 µM. Images taken at 200 × magnification with a scale bar of 100 µM. (**c**) Quantification of *Notch*1 positive cells in the interstitium from the RNAscope staining using Weka Classifier by Fiji ImageJ. Significance ****P < 0.0001, ns, not significant. Data represented as mean ± sem.

PDGFrβ positive pericytes transforming into myofibroblasts is a key component in the development of interstitial fibrosis^29,30^. Based on our network plot analyses at six months (Supplementary Fig. S2), we identified that PDGFrβ expression was reduced with the lithium-amiloride group. This was suggestive that activated pericytes may be recruited and mediate in part the lithium induced interstitial fibrosis and amiloride may down-regulate this activation. To explore this further, we used PDGFrβ immunohistochemistry to examine the role of activated pericytes in this model. Lithium treatment alone demonstrated increased PDGFrβ positive cells in the interstitium around dilated tubules at 28 days and 6 months (Supplementary Fig. S6c and Fig. 6a) with fewer PDGFrβ positive cells in the lithium-amiloride group. Additional marker characterization of PDGFrβ positive cells revealed many were p65^Ser536^ positive and some were also positive for the myofibroblast marker αSMA (Supplementary Fig. S7). These data suggest amiloride exposure reduced the amount of activated pericytes in the kidney interstitium surrounding the dilated tubules.

Transition of PDGFrβ positive pericytes into myofibroblasts has been associated with Notch1^27^. Although *Notch1* was not significantly increased, the *Notch1* ligand Jag1 expression was increased with lithium alone (0.63log2FC) and decreased with lithium-amiloride (− 0.49 log2FC) at 6 months. Increased *Jag1* expression has been found to be dependent upon activation of NF-κB/RelA^31^.

Here lithium alone was associated with increased expression of p65^ser536^ (NF-κB/RelA) (Fig. 4a,b) with increased *Jag1* evident from the network plot (Supplementary Fig. S2a). Therefore, we investigated if *Notch1* expression was increased. The spatial distribution of *Notch1* was determined using RNAscope which was found predominantly in the interstitium with minimum expression in the tubular cells (Fig. 6b). Regions associated with increased *Notch1* expression overlapped with PDGFrβ positive cells, suggesting that these were Notch 1 positive pericytes. Furthermore, the in-situ images demonstrated reduced *Notch 1* positive pericytes with amiloride exposure, further supporting an anti-inflammatory function for amiloride use.

## Discussion

**Figure 7:**
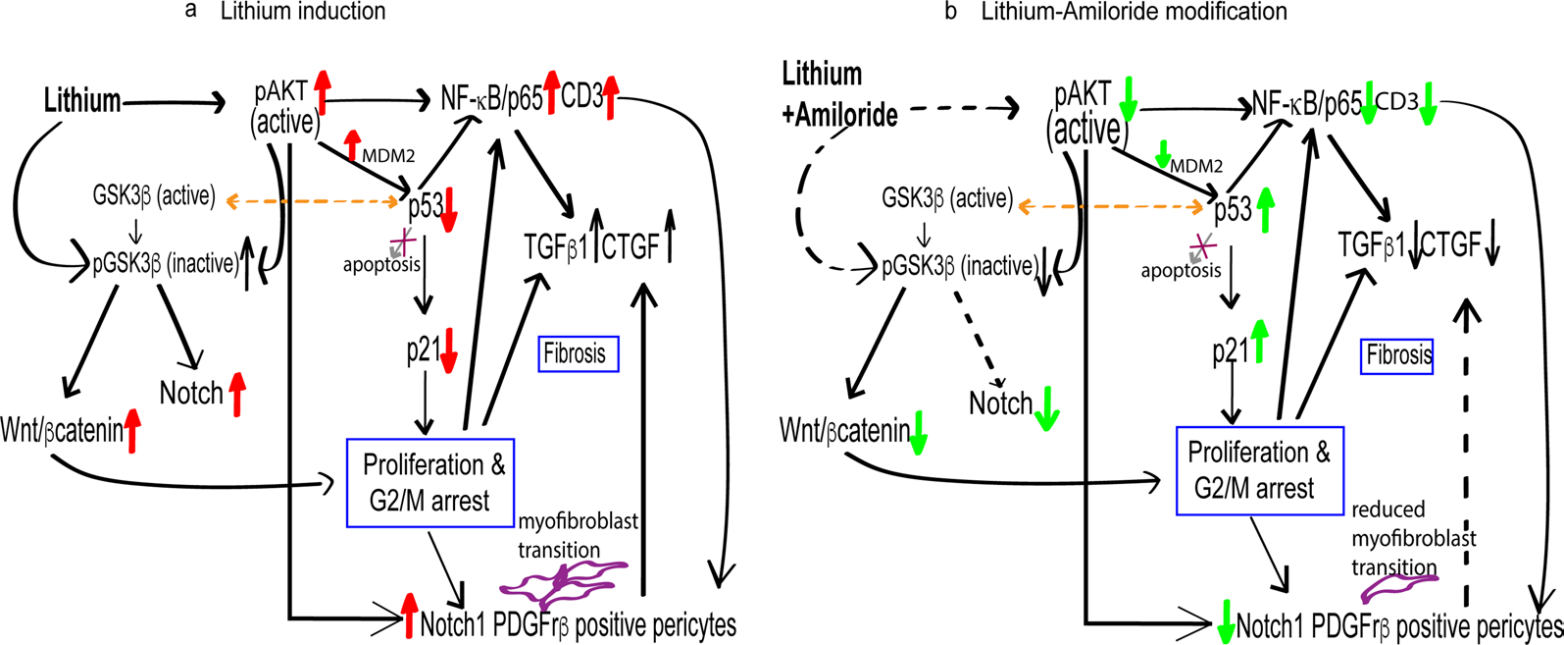
Overview of pathways altered by lithium and amiloride found in this study and supported from the literature. From this study: (**a**) Lithium leads to increased active AKT (pAKT), increased Wnt/βcatenin potentially via increasing levels of phosphorylated pGSK3β (inactive), increased NF-κB/p65, infiltrating CD3 T cells and decreased p53 function via MDM2*. It also promotes PDGFβ production and the recruitment and accumulation of notch1 positive PDGFrβ pericytes leading to increase profibrotic cytokines TGFβ1 and CTGF promoting fibrosis^3^. (**b**) Amiloride administered in combination with lithium reduces active AKT (black dotted line) decreased Wnt/βcatenin potentially via decreasing levels of phosphorylated pGSK3β (inactive), decreased NF-κB/p65 and increased p53 that in turn further negatively regulates NF-κB/p65 with decreased infiltrating CD3 T cells. A robust p53 response results in downstream targets of p53 including p21 to be increased. PDGFrβ production is reduced resulting in reduced Notch1 positive PDGFrβ pericytes in the interstitium and a decrease in profibrotic cytokines TGFβ1 and CTGF (dashed black line). The reduced inflammation leads to reduced fibrosis^3^. From the literature: Lithium directly inactivates constitutively active glycogen synthase kinase 3β (GSK3β) by phosphorylation (pGSK3β)^32^. Inactive pGSK3β activates and increases Wnt/βcatenin signalling that causes cell arrest contributing to pericyte recruitment^38,78^ and fibrosis^39^. Active GSK3β is required for transactivation of p53^41^ (dashed yellow line) and inactive pGSK3β enhances Notch1 activity. Lithium-Amiloride could potentially be reducing the inactive pGSK3β that could lead to reduced expression of Notch1 (dashed black lines)^45,46^. *Data derived through cell culture (not shown, unpublished study).

In this study, we identified genes and pathways involved in lithium induced progressive interstitial fibrosis and how amiloride interacts with these pathways to down-regulate the progression of interstitial fibrosis (Fig. 7). The top candidates identified by RNA sequencing, validated using immunohistochemistry and immunofluorescence, included activated PI3k/Akt, p53 and its downstream target p21, NF-κB/p65^ser536^, and CD3 T cells. Other targets included Notch1 positive PDGFrβ pericytes involved in pericyte to myofibroblast transformation, recently identified as a regulator of kidney fibrosis^30^. On the basis of these results, we highlight molecular targets that are modified by the actions of amiloride in combination with lithium.

Lithium induced interstitial fibrosis is initiated following G2M arrest that is associated with a maladaptive response with persistent inflammation^8,20^. Lithium directly inhibits GSK3β^32^ and indirectly via activation of AKT through PI3k/Akt signalling^33^. Physiologically, GSK3β is constitutively expressed and required for cell cycle turnover^34,35^, but inhibition of GSK3β leads to stabilization of β-catenin, as seen with increased β-catenin expression following lithium treatment in the tubular epithelial cells lining the dilated distal tubules^36,37^. Activated Wnt causes cell arrest in the G2M phase^38^, which is associated with increased interstitial fibrosis^39^ mediated via increased activation of pericytes along with pericyte to myofibroblast transformation^40^. Of interest, we did not observe any changes in GSK3β gene expression. A possible explanation is that as GSK3β is ubiquitously expressed, total GSK3β may not change, rather the intracellular ratio of active GSK3β versus inactive pGSK3β is critical for the observed changes seen. As the role of GSK3β in lithium induced changes are well documented in other studies^36,37^ we focused on the regulatory pathways upstream of GSK3β as well as other linked pathways associated with lithium induced fibrosis.

p53 and NF-κB signalling pathways are both activated under cellular stress and have opposing outcomes with p53 promoting anti-inflammatory responses and NF-κB promoting pro-inflammatory responses^21,22^. p53 expression was increased in dilated tubular epithelial cells suggesting increased stress following lithium exposure. A robust p53 response may be compromised with lithium, given that GSK3β activity is required for p53 function and lithium inhibition of GSK3β would indirectly down-regulate p53 function^41^. In addition, pAKT^ser473^ which was increased in the dilated tubular epithelial cells with lithium, would also down-regulate p53 function by directly interacting with MDM2 leading to ubiquitination of p53^24^. From the RNA seq data, we found *MDM4 at* 6 months and *MDM2* gene expression at 14 days significantly increased with lithium consistent with lithium reducing p53. Other negative regulators of p53, were increased including Tert^42,43^. Given activated pAKT^ser473^ increases *Tert*^44^, upregulation of *Tert* offers another explanation for the reduced p53 function following lithium induced damage.

This study found that co-administration of amiloride significantly reduced inflammation and fibrosis. Amiloride reduced pAKT^ser473^ expression and increased p53 which could explain the decrease in NF-κB/p65 expression, and decreased recruitment of *Notch1* positive PDGFrβ and CD3 T cells in the interstitium. Collectively these changes were associated with reduced inflammation and a decrease in profibrotic cytokines TGFβ1 and CTGF with a marked reduction in interstitial fibrosis^3^. Lithium-amiloride could also potentially reduce pGSK3β^ser9^ (inactive) expression, allowing active GSK3β mediated down-regulation of *Notch1* expression further contributing to the downregulation of activated pericytes as previously demonstrated^45,46^. In this study, we were not able to demonstrate successful GSK3β and pGSK3β^ser9^ co-staining with *Notch1* to further support this conclusion.

Further support for an enhanced role for p53 with amiloride, comes from the lithium-amiloride treatment group having increased microRNA34a expression. This microRNA is transactivated by p53^23^ and together are able to suppress Wnt/βcatenin signalling that has been implicated in kidney fibrosis^47^. Other studies have suggested that miRNA34a is downregulated by Notch1^48^. This also supports the observations seen in this study. Overall, the results suggest that reduced pAKT^ser473^ signalling is an essential component in amiloride-induced reduction in interstitial fibrosis. Increased p53 expression could be potentiated via a feedback loop mediated through PTEN further inhibiting pAKT^49^.

Increased expression of PDGFrβ positive pericyte accumulation is an essential component of interstitial proliferation in response to injury and the development of interstitial fibrosis^50–52^. Notch signalling plays an important role in the development and transition of pericytes into myofibroblasts^27^. Studies have shown active pAKT^ser473^ is essential for activation of Notch signalling^53^. Co-labelling sections with αSMA clearly showed sparse but distinct co-localisation of PDGFrβ postive pericytes to αSMA labeled myofibroblasts in the interstitium. Taken together the co-localisation of *Notch1 and* PDGFrβ (Fig. 6) and pAKT^ser473^ and PDGFrβ expression in interstitial cells (Supplementary Fig. S8) upon lithium exposure supports the concept of Notch-mediated PDGFrβ positive pericytes being recruited to sites of damaged tubules and transitioning into myofibroblasts mediated via active pAKT^ser473^ in response to chronic lithium exposure.

Co-administration of amiloride with lithium from day 0 did not prevent the changes in cell proliferation and G2M arrest leading to the dilated cystic tubules that were evident at 14 and 28 days. Lithium at therapeutic doses is thought to suppress GSK3β activity by approximately 25%^35,54^. So clearly in this model there is not complete inhibition of GSK3β activity. Amiloride would reduce cellular uptake of lithium via inhibition of sodium channels and therefore potentially reduce intracellular lithium concentrations which may allow a reduction in GSK3β inhibition. This was observed with increase in p53, p21, reduced *Notch1* and reduced NF-κB/p65 mediated inflammation that was evident from as early as day 14 with amiloride co-treatment. However, clearly amiloride did not counteract all of the pathways associated with lithium induced cellular injury, especially those related to tubular epithelial cell proliferation and subsequent G2/M arrest^6–8^.

Activated p53 can lead to induction of downstream target genes including *cdkn1a/p21* and *Bax*, and depending on the stimulus, causes either cell arrest^55^ or apoptosis^56^ respectively. Following short term (4–13 days) lithium treatment, de Groot et al.^8^ demonstrated that proliferating principal cells were arrested at the G2/M phase which may contribute towards fibrosis^8^. In the current study, lithium alone and lithium-amiloride was associated with increased p21 expression compared to controls. In other models of kidney fibrosis, an increase in p21 expression has been linked to G2/M cell arrest^57^. This would suggest that lithium is having a direct effect on p21 expression which is not modified by amiloride. The proliferating tubular epithelial cells arrested at the G2/M phase, following lithium induced increased expression of p21, leads to a pro-inflammatory state with activation of NF-κB/p65 mediated inflammation and increased expression of pro-fibrotic cytokines^39^. However, the robust increase in p53 expression as well as the observed increased p21 expression in the dilated tubular epithelial cells in the lithium and amiloride, may in part explain^8^ the reduced fibrosis when compared to the lithium alone group.

Tumor suppressor p53 regulates the cell cycle and the arrest after cell lineage decision is made via Notch signalling^58^. Lineage decision, inductive signalling and later inhibition are the main functions of Notch signalling^59^. Active Notch favors principal cell development, whereas inactive Notch favors intercalated cell development^60^. Lefort et al., using a keratinocyte cancer and squamous cell carcinoma lines, showed that *Notch1* is a target of p53 with an active role in tumor suppression^61^. Lithium induced tubular injury is associated with principal cells de-differentiating to intercalated cells^6,7,62,63^. Increased *Foxi1* transcription, a key marker of intercalated cells^60^ with lithium treatment was significantly and differentially expressed at 28 days and 6 months (0.94 and 1.05 log 2FC, respectively) (Supplementary Dataset 1). Given lithium treatment can alter the ratio of principal to intercalated cells, it is not surprising that in this study *Notch1* expression was predominantly seen in the interstitium and not in the tubular epithelial cells due to the reduction of principal cells. Notch1 signalling has also been implicated in enhancing fibrosis and its inhibition rescuing fibrosis^64^.

In conclusion, the previously demonstrated lithium induced overexpression of number of intracellular pathways including ‘cell cycle signalling’, ‘NF-κB signalling’, ‘p53 signalling’, ‘Wnt signalling’ and ‘aldosterone up-regulated genes’^9^ was confirmed at later time points of 14 days, 28 days and at 6 months. Combined treatment with amiloride, significantly downregulated the expression of those gene pathways associated with inflammation resulting in a marked reduction in interstitial fibrosis. The interaction of these pathways are summarized in Fig. 7. However, the lithium induced cell proliferation and G2/M growth arrest which is associated with dilated distal tubules and altered tubular epithelial cells, was not modified by amiloride. This would suggest that amiloride does not modify ‘cell cycle signalling’ pathways directly. Further studies are underway to examine the anti-inflammatory actions of amiloride.

## Methods

### Kidney fibrosis animal model

Rat kidney cortical tissue from our previously published long-term (6 months) animal model^2,3^ was used here alongside rat kidney cortical tissue from short term lithium treated animals (14 and 28 days). Animals were fed lithium for 6 months and co-administered amiloride for five months that maintained plasma lithium levels equivalent to that used therapeutically^2,3,65^. The animal studies were performed in accordance with ARRIVE guidelines.

Short term animal study for 14 and 28 days included male Wistar rats (~ 200 g) from the Hercus-Taieri Resource Unit, University of Otago that were separated into three groups; Control n = 6, lithium alone (Li, n = 6), and lithium with amiloride (LiAM, n = 6). Here animals were treated following the protocol from our previous study but were instead given amiloride from day zero along with lithium. Anesthetised animals were euthanized using decapitation (guillotine). Excised kidney tissue was either frozen and stored at − 80 °C in RNAlater®, or fixed in 10% neutral buffered formalin (NBF) and processed into formalin fixed paraffin embedded (FFPE) blocks for downstream analysis. Ethical approval for the protocols described here was given by the University of Otago Animal Ethics Committee (81/14), under New Zealand National Animal Welfare guidelines.

To induce chronic interstitial fibrosis, the protocol from Croft et al. was modified^3^. The diets were administered as follows: control rats were given a normal chow diet (Speciality Foods, Perth, Australia), lithium alone group received 40 mmol lithium/kg dry food for the first 7 days, followed by 60 mmol lithium/kg dry food (lithium diet) for up to 14 or 28 days and the LiAM group were given standard chow with lithium and amiloride drinking water containing 0.2 mmol/l amiloride (Sigma Aldrich, USA). This protocol results in plasma lithium levels comparable to therapeutic levels in human plasma (0.8–1.3 mmol/l) and minimizes the reduced weight gain that can be caused by lithium. To enable adequate mixing with the drug, all pellets were pulverized with a kitchen blender, sifted, ground into a fine powder in a small blender and sifted again. All rats administered lithium received a salt block to maintain sodium balance and to prevent lithium intoxication. Water and food intake was measured every day and body weight every other day. For the last two days of the experiment, rats were housed individually in metabolic cages on a 12:12 h light–dark cycle and allowed to settle for 21 h.

### Blood plasma and urine analysis

Blood and urine analyses were performed to characterize the effect of lithium and amiloride on kidney function. The analyses for the six-month study were performed and published previously^3^. For the 14- and 28-day studies the osmolality of urine and plasma was measured using Wescor 5500 Vapro® Osmometer (Logan, UT, USA). Sodium and potassium ion concentrations were determined by flame photometry (SEAC FP20, Italy), and chloride ions in the plasma were determined electrometrically using a Cotlove chloride titrator (American Instrument Co., Silver Spring, MD, USA). Lithium-ion concentration in plasma was measured with a contrAA® 700 High-Resolution Continuum Source atomic absorption spectrometer (Analytik Jena AG, Germany). Creatinine in urine was measured according to Jaffe’s method (Pointe Scientific Inc., Canton, MI, USA), and urea (blood urea nitrogen (BUN); Pointe Scientific Inc., Canton, MI, USA) was measured in plasma. Total urinary protein was determined with a bicinchoninic acid (BCA) assay (Pierce Chemical Co., ThermoFisher Scientific, Rockford, IL, USA). Blood and urine analysis are given in Supplementary Table 1.

### Quantifying interstitial fibrosis

Kidney sections of 4 µm stained with Masson’s trichrome (MST) were used to estimate the percentage of collagen deposition indicative of interstitial fibrosis. Aperio Imagescope (Aperio, Vista, California, USA) software at × 200 magnification was used with the grid function. From ten random fields around the cortex, 4000 grids per field were made and MST staining in areas around the dilated tubules or tubules in the cortex of control group was counted. Thin layer of staining around the vessels was excluded. Positive number of squares per field were divided from 4000 grids per field to get the percent positive area of interstitial fibrosis.

### RNA extraction and quantification

RNA was isolated from the cortical region of the frozen kidney using TRIzol™ (Thermo Fisher Scientific), and the Total RNA Purification Kit (Norgen Biotek Corp, Ontario, Canada). RNA was quantified using NanoPhotometer N60/N50 spectrophotometer (IMPLEN, CA, USA) and integrity verified using RNA 600 Nano 6000 Assay kit with Bioanalyzer 2100 system (Agilent Technologies, CA, USA).

### RNA sequencing

Library generation for RNA sequencing was performed using Illumina TruSeq Stranded mRNA IDT for Illumina Unique Dual Indexing then run on the HiSeq 2500 V2 Rapid sequencing platform with Single end 100 base pair reads at the Otago Genomics Facility. Raw data was subject to Quality Control (QC) using FastQC version 0.11.9. Adaptor sequences and low quality sequences (Phred score < 30) were trimmed using Cutadapt version 2.6^66^. Reads were aligned against the rat reference genome (*Ensembl Rnor6.0*) using HISAT2 version 2.1.0^67^ and annotated with *Rnor 6.0.85.gtf* from *Ensembl*^68^. Aligned reads were counted by exon and summarized by gene using featureCounts version v1.5.3^69^. Counts were normalized for differential gene expression using Wald Test from DESeq2 package (version 1.30.1)^70^ performed locally on R (version 4.0.2) and RStudio™ (version 1.3.1093)^71^. *P*.values were adjusted for multiple testing using Benjamini Hochberg correction. Genes were considered statistically differentially expressed if the criteria of False Discovery Rate (FDR) < 0.05 and log2 fold change of ± 0.5 (FC ± 0.5) were satisfied. Overrepresentation of ontologies and signalling pathways were derived using Metascape^72^ and NetworkAnalyst^73^. Genes of interest were selected based on function and validated using immunohistochemistry. MicroRNA data derived using nCounter Rat miRNA Assay Kit (cat#GXA-RMIR-12) normalized using NanoStriDE for pre-analysis^74^ and NanostringNorm^75^.

### Immunohistochemistry and immunofluorescence

Kidney sections (4 µm) from FFPE tissue blocks were subjected to immunohistochemistry and immunofluorescence (IHC) on a fully automated system (Leica Bond RXm, Leica Biosystems, Illinois, USA) according to the manufacturer’s instructions. Antigen retrieval treatment and antibody dilution was optimized for each antibody (Supplementary Table 2). Slides were then scanned using Aperio Scanscope CS digital pathology system (Aperio, Vista, California, USA). Stained slides were analyzed for total and positive nuclear staining for p53, p21, p65^Ser536^ and cytoplasmic staining for pAKT^Ser473^. This was performed by randomly selecting ten sections within the kidney cortex tissue at 200 × magnification. Positive and total number of cells were measured using cell counter tool on Fiji by ImageJ. Fluorescent staining was performed using the OPAL 7-Color Kit for Multiplex IHC (Akoya Biosciences, Massachusetts, USA). Multiplex staining using fluorophores OPAL 520, 570, 620, 690 and spectral DAPI was performed using automated staining. All fluorophores were used at a dilution of 1:100. Slides were mounted using ProLong™ Gold Antifade Mountant (Thermo Scientific) and scanned using Aperio VERSA digital pathology system (Leica Biosystems). Slide images were analyzed using Fiji by ImageJ software^76^. Quantification of β catenin signal was performed using the Membrane v9 algorithm by Aperio Technologies (Vista, California). Intense positive signals from β catenin expression was scored as 3 + by the membrane v9 algorithm. Percentage of tubules with a score of 3 + positive tubules were used to graphing.

### RNAscope: in situ hybridization

RNAscope 2.5 HD Assay-BROWN manual kit™ (Advanced Cell Diagnostics, Newark, CA, USA) was used to measure custom designed probe for *Notch1* specific to *Rattus Norvegicus* (Ref: 847361), positive control Rn-UbC (*Ubiquitin C*) and negative control probe (the bacterial gene *DapB*) on FFPE serial tissue sections (4 µm). The manufacturer’s protocol for the 2.0 HD Detection Kit (BROWN) assay for kidney tissue was followed. Slides were scanned using Aperio VERSA digital pathology system (Leica Biosystems). Semi-quantification was performed using trainable Weka segmentation for use with RNAscope in situ Hybridization on ImageJ^77^.

### Statistical analysis

One-way ANOVA followed by Tukey’s multiple comparisons test was performed using GraphPad Prism 9.0.0 (GraphPad Software, California USA, Version 9.4.1(458), https://www.graphpad.com/scientific-software/prism/). A *P* value less than 0.05 (*P* < 0.05) was considered significantly different.

## Supplementary Information

Supplementary Information 1.

Supplementary Information 2.

Supplementary Information 3.

Supplementary Information 4.

## Data Availability

Raw files for RNA sequences (FASTQ format) and Nanostring data are deposited in Gene Expression Omnibus with accessions numbers: GSE178950 and GSE178695, respectively.
