# Supplementary material for: Reduction of lithium induced interstitial fibrosis on co-administration with amiloride
Source: Sci Rep. 2022 Aug 26;12:14598. doi: 10.1038/s41598-022-18825-1 (PMC9418221; doi:10.1038/s41598-022-18825-1)
Supplement: Supplementary file 1 — Supplementary Information 1. [file 41598_2022_18825_MOESM1_ESM.pdf]

Supplementary Material

Supplementary Table 1: Plasma and urine physiological parameters of rats treated for 14 days or 28 days with normal food (CTR) or with lithium (Li), lithium + amiloride (LiAM). Values are represented as means ± SE. All *P* values are compared to CTR.

Treatment	*CTR*		*Li*		*LiAM*	
Duration	14d	28d	14d	28d	14d	28d
Plasma composition						
Na^+, mmol/l	144 ± 1	141 ± 1	141 ± 1	138 ± 1	145 ± 2	145 ± 8
K^+, mmol/l	6.4 ± 0.2	5.5 ± 0.3	5.5 ± 0.3	6.0 ± 0.3	6.7 ± 0.4	6.9 ± 0.5
Li^+, mmol/l	/	/	1.72 ± 0.09	1.37 ± 0.08	1.72 ± 0.15	1.46 ± 0.14
Cl^-, mmol/l	100.3 ± 1.9	93.7 ± 1.3	99.6 ± 3.5	100.4 ± 3.3	100.9 ± 2.2	102.9 ± 3.1
Osmotic pressure, mosmol/kgH2O	305 ± 3.4	297 ± 5.3	303 ± 2.7	301 ± 4.6	307 ± 1.6	300 ± 6.3
Urine composition						
Urine output, µl/min/kg BW	3 ± 1	5 ± 1	461 ± 24**	643 ± 40**	189 ±24*	209 ± 24*
Na^+, mmol/l	120 ± 11	184 ± 18	19 ± 2	21 ± 2	157 ± 20	150 ± 9
K^+, mmol/l	303 ± 21.8	406± 47.7	14 ± 0.5	12 ± 0.6	41 ± 3.6	36 ± 4.6
Cl^-, mmol/l	174.6 ± 23.1	253.5 ± 13.9	25.0 ± 3.3	26.0 ± 3.2	118.6 ± 16.2	133.1 ± 7.8
Protein, mg/h/100g	1.07 ± 0.32	1.34 ± 0.20	3.03 ± 0.04	3.60 ± 0.47	4.15 ± 0.09	3.13 ± 0.27
Total Na^+ excretion (mmol/24h)	0.48 ± 0.17	0.77 ± 0.16	2.49 ± 0.19	5.67 ± 0.77	8.87 ± 1.05	12.27 ± 1.55
Total K^+ excretion (mmol/24h)	1.21 ± 0.42	1.62 ± 0.21	1.82 ± 0.07	3.08 ± 0.24	2.29 ± 0.14	2.78 ± 0.24
Total Cl^- excretion (mmol/24h)	0.48 ± 0.16	1.08 ± 0.25	3.28 ± 0.36	7.05 ± 1.02	6.48 ± 0.66	10.79 ± 1.29

* $P<0.05$

** $P<0.01$

/ not determined

Supplementary Table 2: Antigen retrieval and antibody dilutions used for immunohistochemistry

**Name**	**Clone**	**catalogue number and Company**	**Heat-induced epitope retrieval (ER) solution and time (mins)***	**Dilution**
(Active) β-Catenin (Ser33/37/Thr41)	D13A1	Cell Signalling Technologies (#8814)	ER 2, 20 mins	1/1500
Phospho-NF-κB p65 (Ser536)	93H1	Cell Signalling Technologies (#3033) (Massachusetts, USA)	ER 1, 20 mins	1/200
CD3	-	Abcam (ab5960)	ER 1, 20 mins	1/100
p53	Sp5	Cell Marque (California, USA)	ER 2, 20 mins	1/50
p21	EP147	Cell Marque	ER 2, 20 mins	1/50
Phospho-Akt (Ser473)	D9E	Cell Signalling Technologies (#4060)	ER 2, 20 mins	1/100
PDGFR beta - C-terminal	Y92	Abcam (ab32570)	ER 2, 20 mins	1/1000
alpha smooth muscle actin	E184	Abcam (ab184705)	ER 2, 20 mins	1/1000

***Solutions from Leica Biosystems**

Supplementary Figures

Supplementary Figure S1

a Principal component analysis of all samples from 6 months

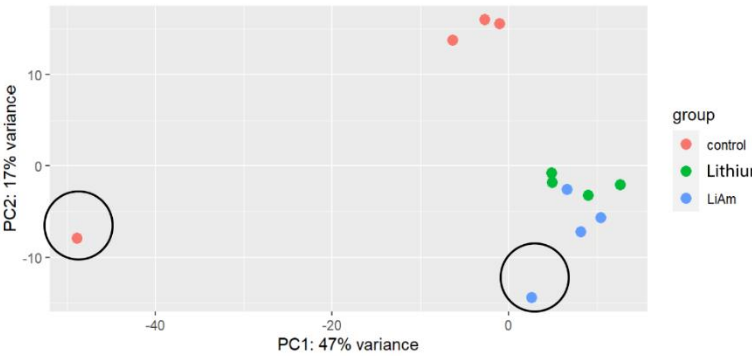

b Principal component analysis of all samples from 14 days

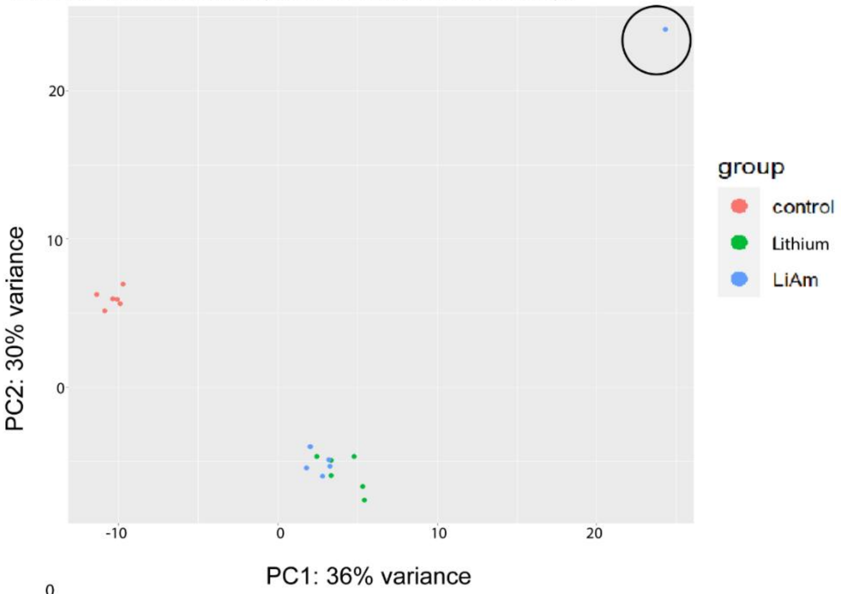

0

c Principal component analysis of all samples from 28 days

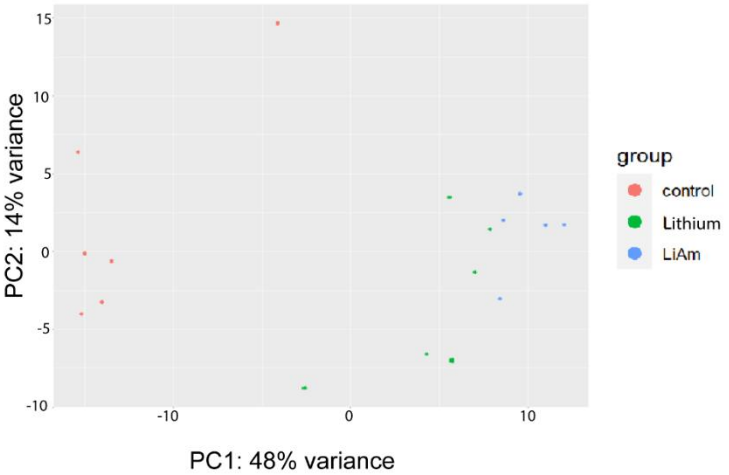

Supplementary Figure S1. Principal component analysis of all samples including outliers from **a)** 6 months and **b)** 14 days including the outliers (circled) and **c**) at 28 days with no outliers.

Supplementary Figure S2

a Lithium/Control group

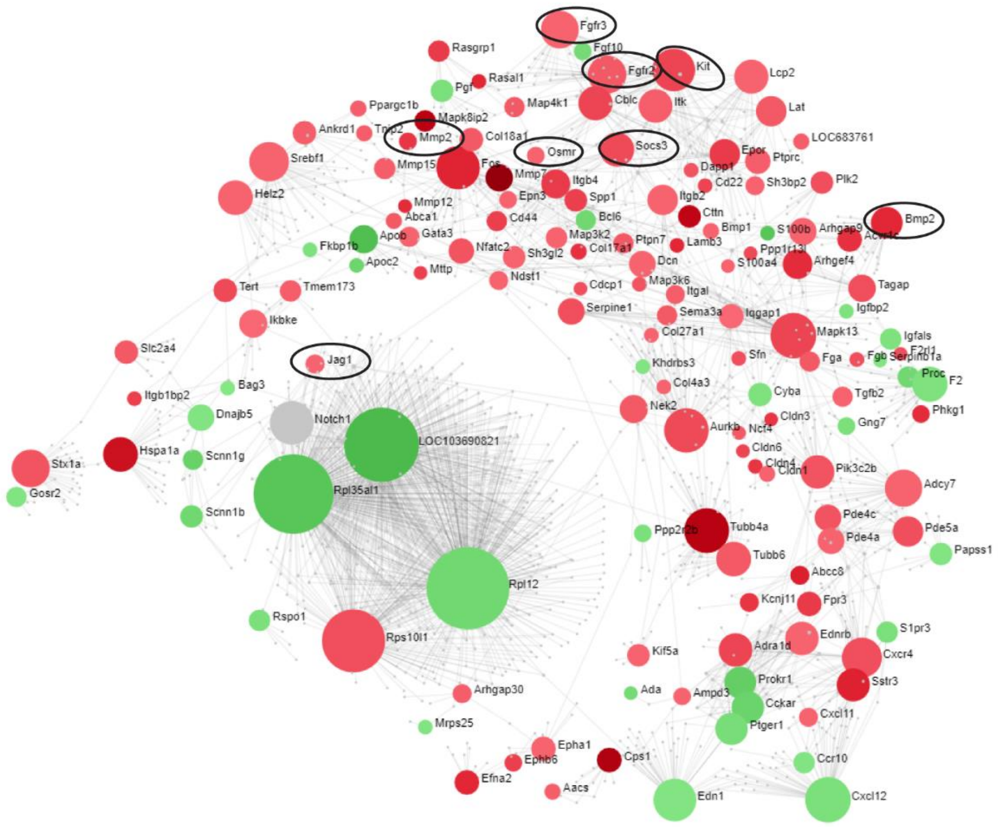

b LithiumAmiloride/Lithium group

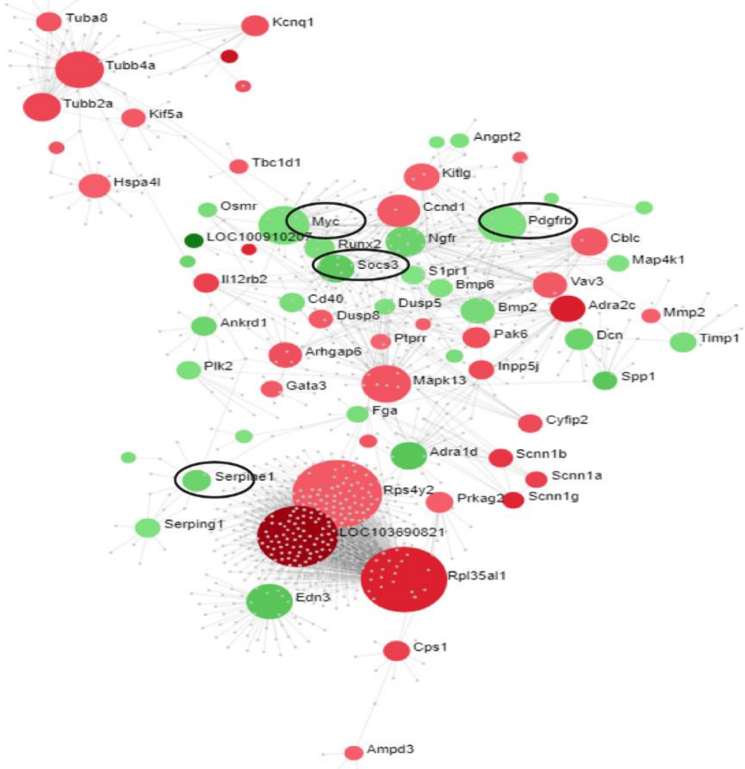

Supplementary Figure S2: Network analysis of differential gene expression of **a)** lithium versus controls. **b)** LithiumAmiloride versus lithium alone.

Supplementary Figure S3

a Network of genes in Lithium/Control at 14 days (n=1807)

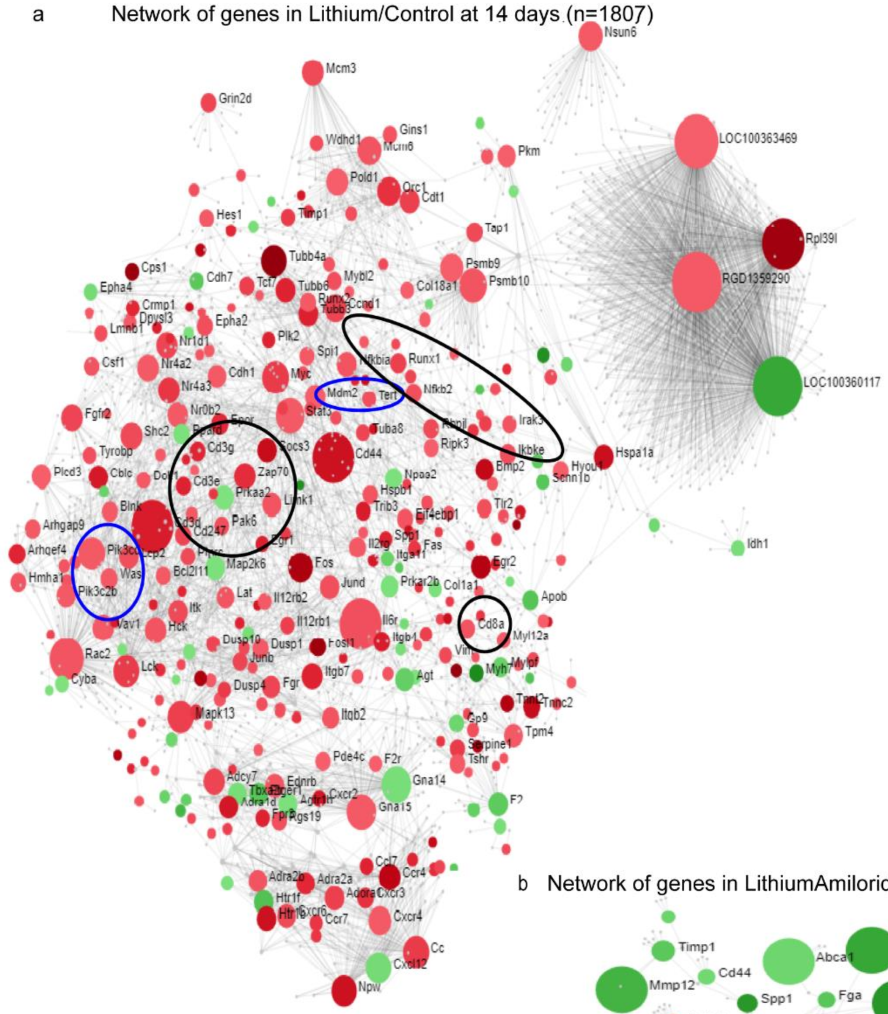

b Network of genes in LithiumAmiloride/Lithium at 14 days (n=439)

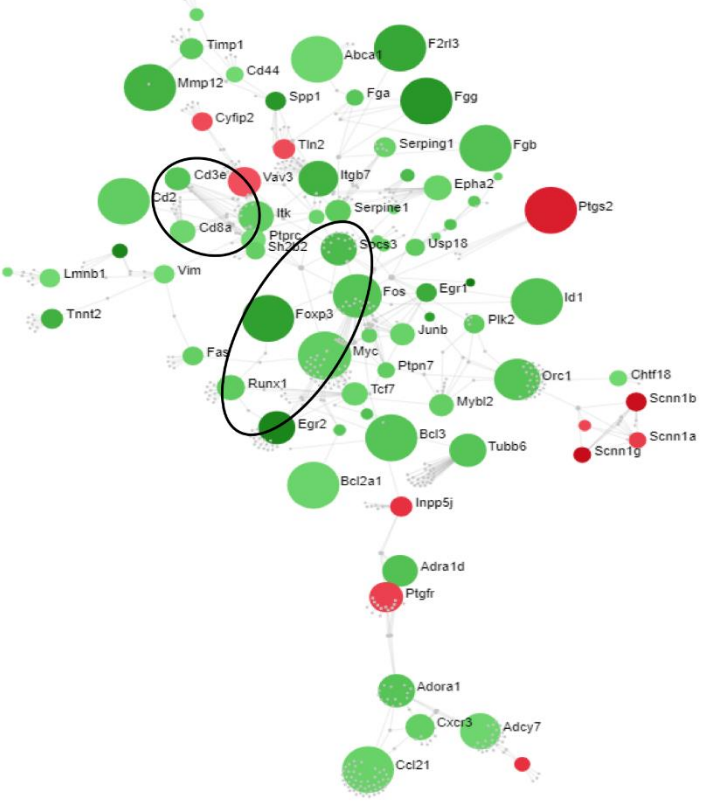

Supplementary Figure S3: Network analysis of from differential gene expression of **a)** Lithium/control and **b)** LithiumAmiloride/Lithium at 14 days.

Supplementary Figure S4

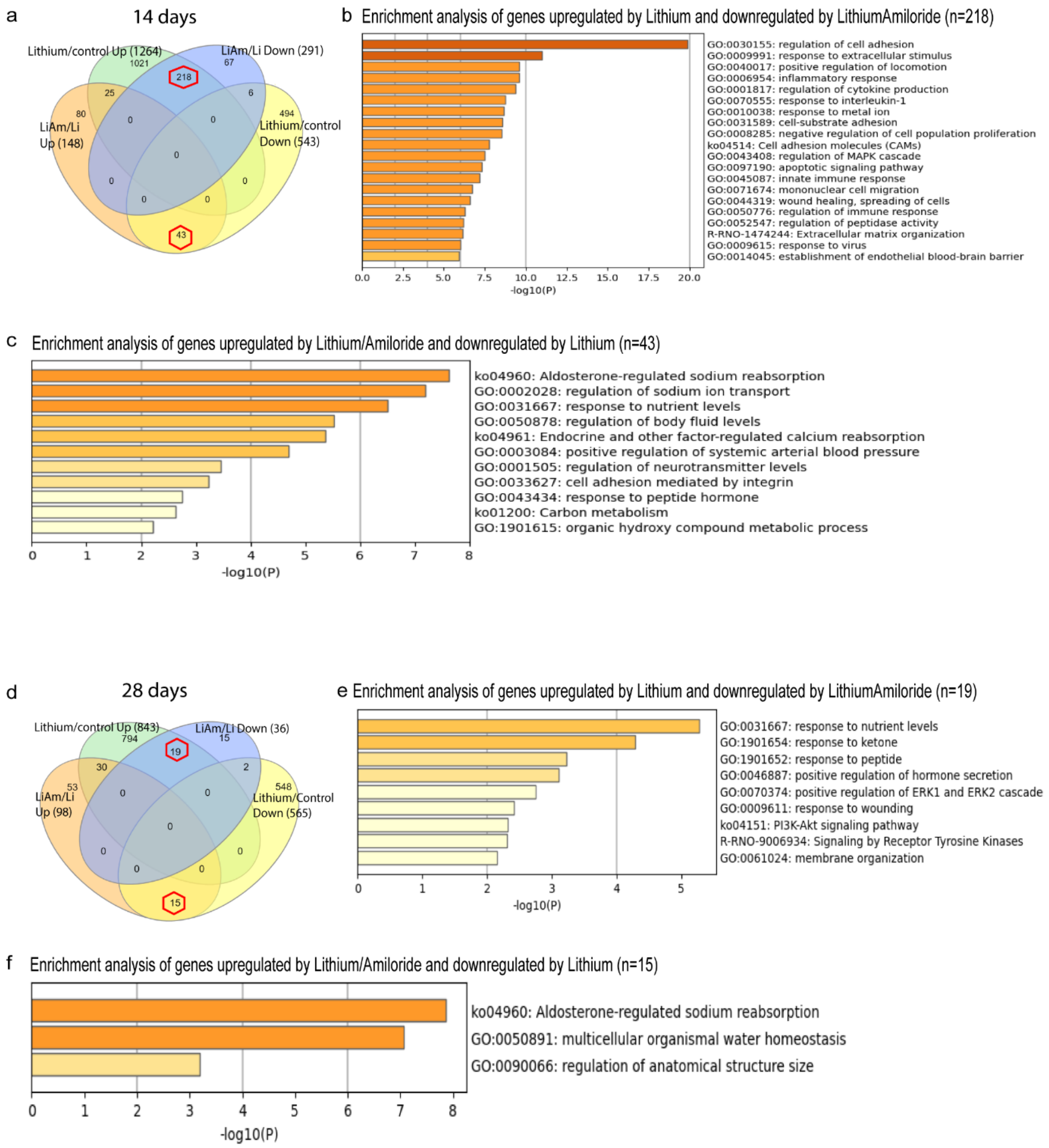

Supplementary Figure S4: Overrepresentation analysis of overlapping genes with FDR (<0.05), log2FC threshold (±0.5) at 14 and 28 days. **a)** Venn Diagram of 14 days: Total genes

in Li/Control (n=1087, increased n= 1264 and decreased n=543), LiAM/Li group (n=439, increased n= 148 and decreased n=291). **b**, **c)** Over representation of ontologies associated with statistically significant genes upregulated with lithium and downregulated with LithiumAmiloride at 14days (n=218) and 28 days (n=19), respectively. **e**, **f)** Over representation of ontologies associated with statistically significant genes upregulated with LithiumAmiloride and downregulated with Lithium at 14 days (n=43) and 28 days (n=15) respectively. **d)** Venn Diagram of 28 days: Total genes in Lithium/control (n=1408, increased n=843 and decreased n=565), LiAM/Li (n=134, increased n=98 and decreased n=36).

Supplementary Figure S5

MicroRNA data from 6 month study

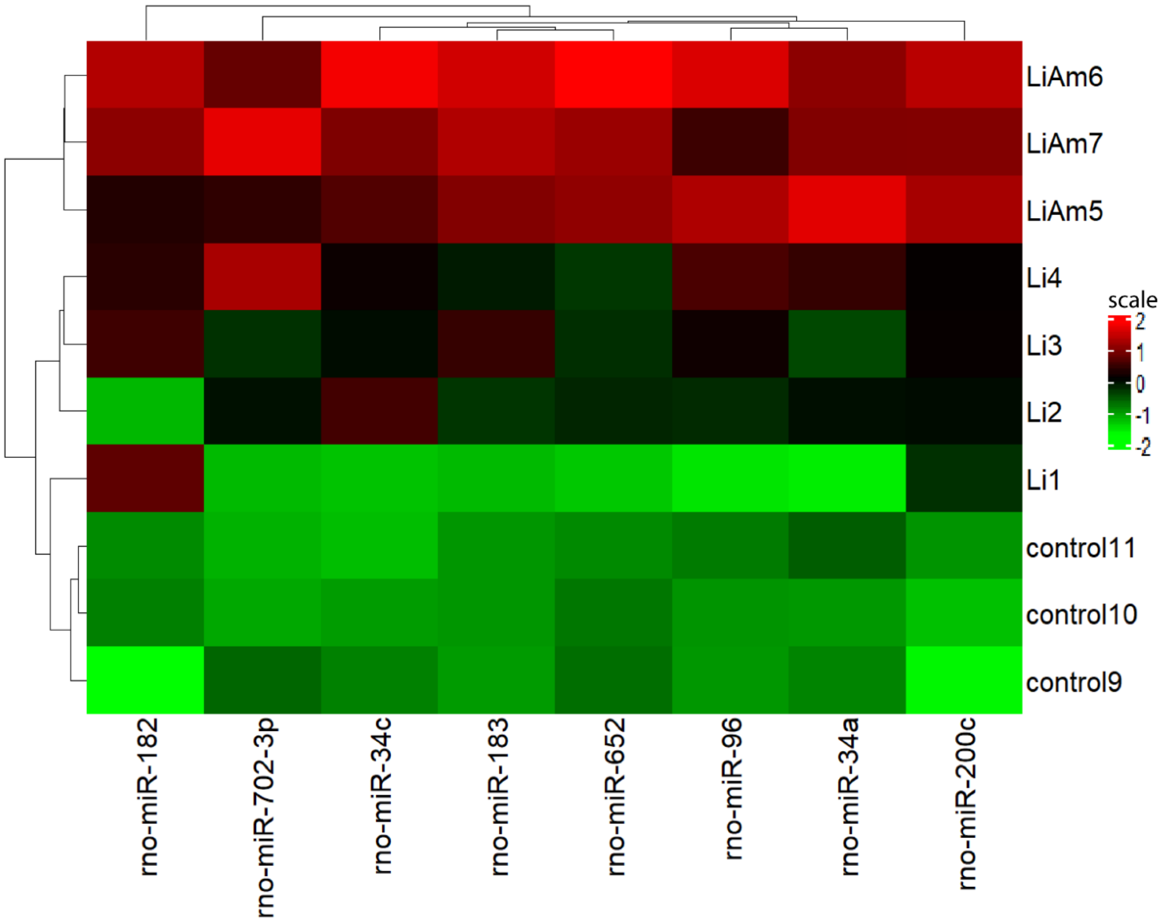

Supplementary Figure S5: MicroRNA expression data from 6 months study. Li, lithium; LiAm, lithium and amiloride and controls.

Supplementary Figure S6

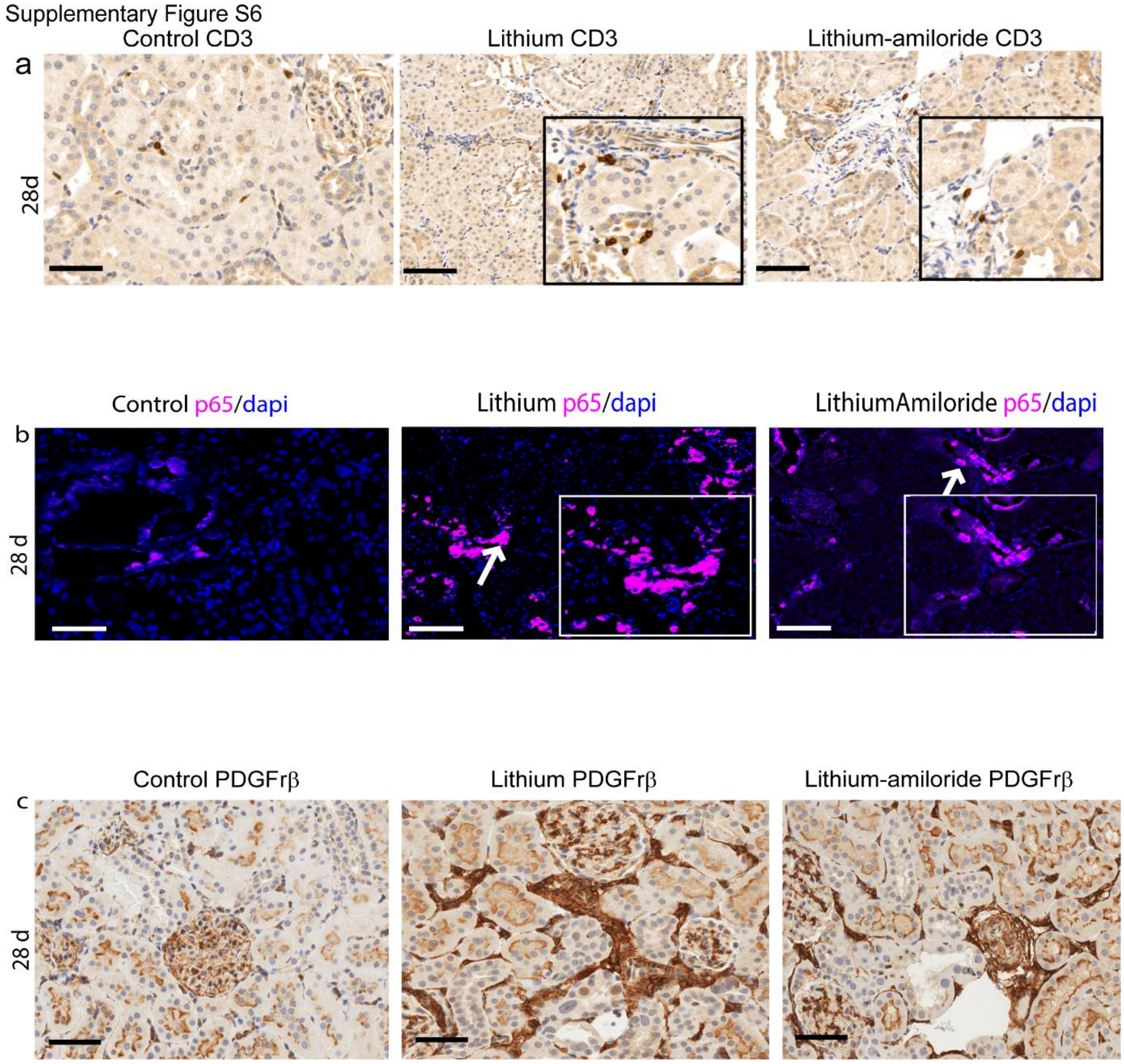

Supplementary Figure S6: Interstitial space and tubular epithelial cells labelled with a) CD3 , b) p65(NFkB) and c) PDGFrB cells at 28 days. Lithium samples had increased infiltration of CD3 positive cells and PDGFrB positive pericytes in the interstitium compared to LithiumAmiloride treated samples. PDGFrB positive pericyte activation at 28 days showss collagen-producing cells involved in the early stages of fibrosis. NF-kB/p65 positive tubular epithelial cells were increased in the lithium samples.

Supplementary Figure S7

a

aSMA/dapi

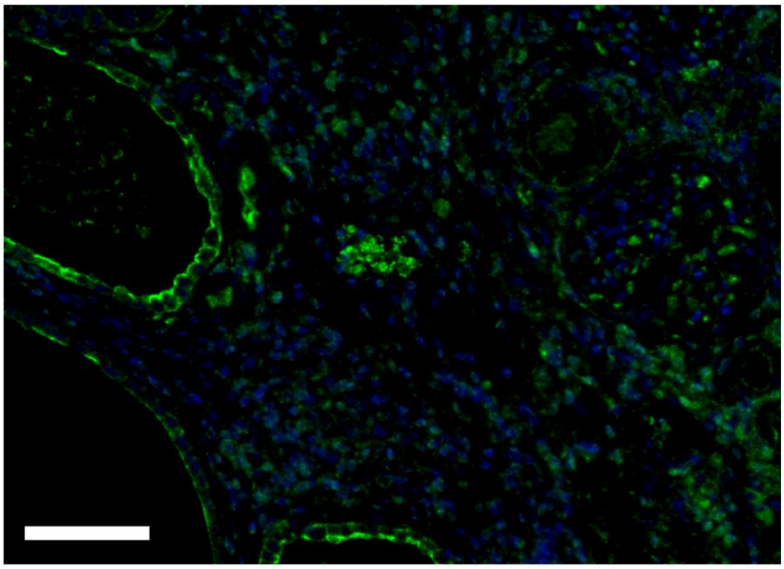

b

PDGFrb/dapi

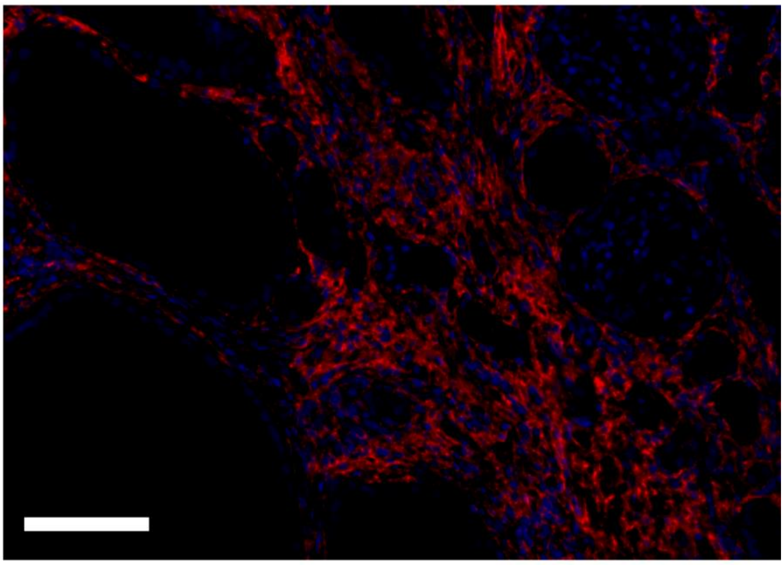

c

SMAa/PDGFrb/dapi

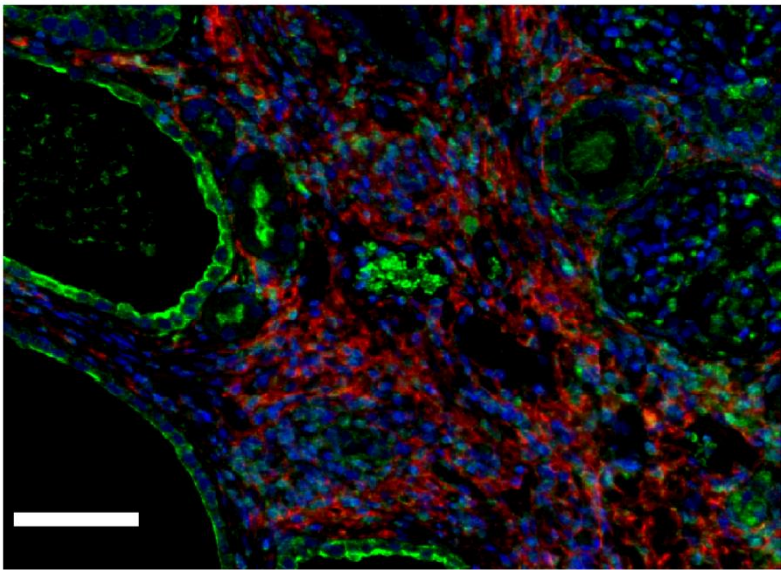

Supplementary Figure S7: Co-localization of αSMA and PDGFRβ on lithium treated kidneys at six months.

Supplementary Figure S8

PDGFrb/pAKT/dapi Lithium

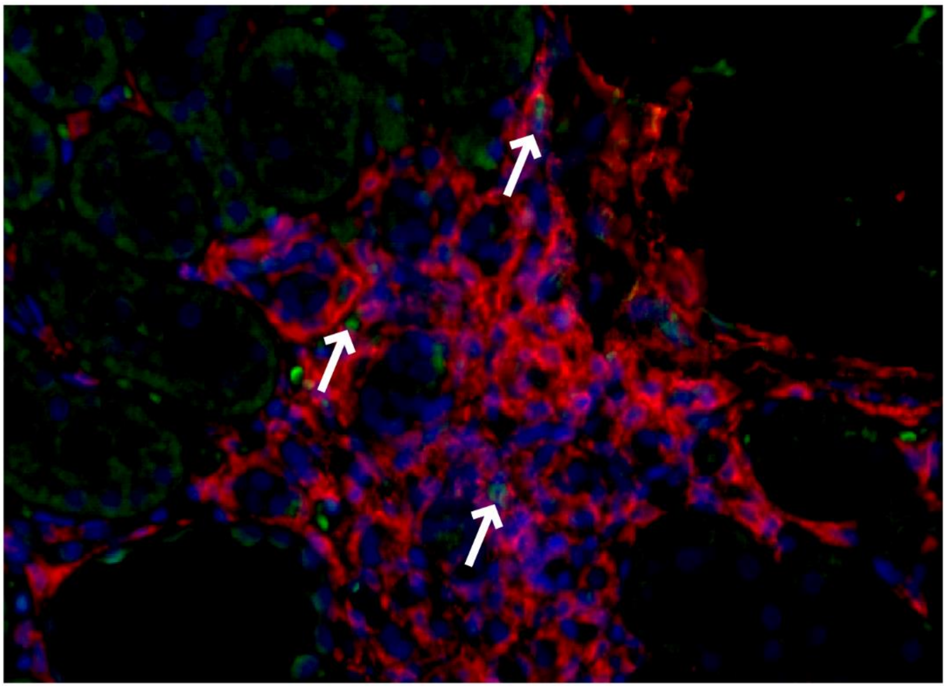

pAKT Lithium

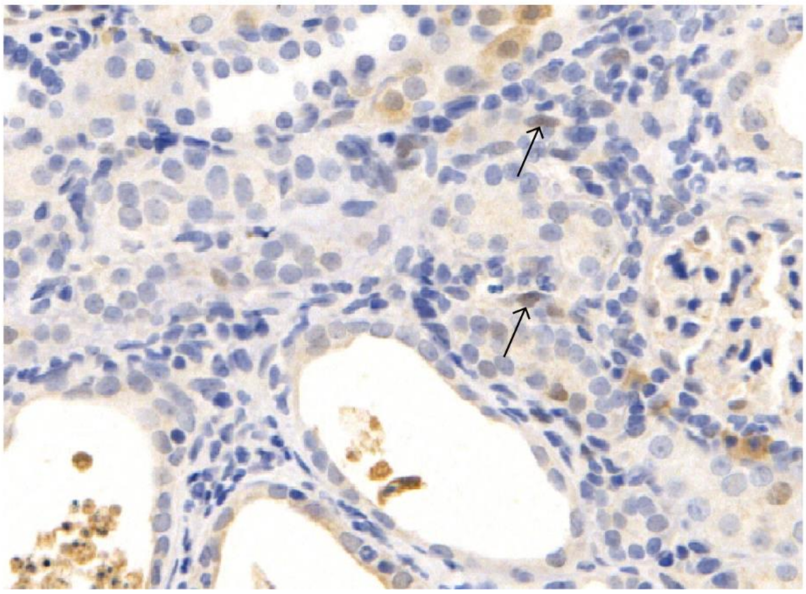

Supplementary Figure S8: Co-localization of pAKT and PDGFRβ in the interstitium on lithium treated kidneys at six months
